# Factors associated with the development of Congenital Zika Syndrome: a case-control study

**DOI:** 10.1186/s12879-019-3908-4

**Published:** 2019-03-22

**Authors:** Giulia P. Lima, Daniel Rozenbaum, Clarisse Pimentel, Ana Cristina Cisne Frota, Daniela Vivacqua, Elizabeth S. Machado, Fernanda Sztajnbok, Thalita Abreu, Raquel A. Soares, Cristina B. Hofer

**Affiliations:** Instituto de Puericultura e Pediatria Martagão Gesteira, Universidade Federal do Rio de Janeiro, R Bruno Lobo, 50, Ilha do Fundao, Rio de Janeiro, Brazil

**Keywords:** Congenital Zika Syndrome, Rio de Janeiro, Case-control, Gestational age

## Abstract

**Background:**

We aim to investigate possible maternal- and pregnancy-related factors associated with the development of Congenital Zika Syndrome (CZS) in children of mothers with probable gestational infection.

**Methods:**

This case-control study, we recruited mother-infant pairs between May 2015 and October 2017 in a pediatric infectious disease clinic in Rio de Janeiro. Inclusion criteria required either that the mother reported Zika infection symptoms during pregnancy or that the infant presented with clinical or imaging features of the CZS. Exclusion criteria included detection of an alternative cause for the patient’s presentation or negative polymerase chain reaction assays for Zika in all specimens tested within 12 days from the beginning of maternal symptoms. Infants with CZS (CDC definition) were selected as cases and infants without CZS, but with probable maternal Zika virus infection during pregnancy, were selected as controls. Maternal and pregnancy-related informations were collected and their relationship to the presence of congenital anomalies due to CZS was assessed by Fisher exact or Mann-Whitney test.

**Results:**

Out of the 42 included neonates, 24 (57.1%) were diagnosed with CZS (cases). The mean maternal age at the birth was 21 years old. The early occurrence of maternal symptoms during pregnancy was the only variable associated with CZS (odds ratio = 0.87, 95% CI: 0.78–0.97).

Case’s mothers presented symptoms until the 25th week of gestational age (GA), while control’s mothers presented until 36th weeks of GA. Income; illicit drug, alcohol, or tobacco use during pregnancy; other infections during pregnancy (including previous dengue infection) were not associated with CZS.

**Conclusions:**

Our study corroborates the hypothesis that Zika virus infection earlier in pregnancy is a risk factor to the occurrence of congenital anomalies in their fetuses.

## Background

On March 31, 2016, the World Health Organization published a statement on the evidence for a causal link between Zika virus infection during pregnancy and the occurrence of congenital brain abnormalities, including microcephaly [1]. With further observational studies, a whole set of congenital abnormalities was identified and linked to in utero Zika virus infection. This set of abnormalities was termed the Congenital Zika Syndrome (CZS) and may include, in addition to microcephaly, craniofacial disproportion, irritability, spasticity, seizures, feeding difficulties, ocular abnormalities, and hearing loss on examination, as well as calcifications, cortical disorders, and ventriculomegaly on neuroimaging. Up until October 2017, there were 3689 confirmed cases of the congenital syndrome associated with Zika virus infection in the Americas, most of them in developing countries and affecting the most socially vulnerable groups [2–4]. Despite the substantial reduction in the number of new cases of Zika virus infection, it is important that preventive measures be drawn due to the high prevalence of *Aedes aegypti* mosquitoes in several areas and the possibility of recurrent waves of infection, as has been observed with other arboviruses in other locations.

It has been estimated that 5 to 42% of neonates from infected pregnant women develop congenital abnormalities [2, 5–8]. Nevertheless, there is only a paucity of studies specifically designed to elucidate which maternal- and pregnancy-related factors may be associated with the development of congenital abnormalities among infected pregnant women [9–11]. The identification of such factors might allow the development of interventions aiming to prevent the Congenital Zika Syndrome (CZS). We, therefore, conducted a case-control study to investigate potential risk factors for the development of CZS in neonates from infected pregnant women. We hypothesized that factors associated with the prenatal period (co-infections, trimester of Zika virus infection, and maternal health variables) would be associated with development of CZS.

## Methods

### Study design

This is a nested case-control study. Cases were defined as infants with CZS and controls as infants without CZS. Both possibly exposed to Zika virus during antenatal period.

### Study population

Recruitment occurred between April 2015 and October 2017 at the Instituto de Puericultura e Pediatria Martagão Gesteira’s (IPPMG) pediatric infectious disease clinic, Rio de Janeiro, Brazil. We enrolled mother-infant pairs if they satisfied at least one of two criteria: 1) the mother had a history of gestational symptoms compatible with Zika virus infection, defined as a skin rash with or without other characteristic findings (e.g., fever, rash, conjunctivitis, and/or arthralgia), or 2) the infant was referred to our clinic due to clinical and/or radiologic findings of CZS. The CZS was defined as: severe microcephaly (> 3 SD below the mean), brain abnormalities, ocular findings, congenital contractures, neurological impairments. Patients were excluded if they failed to provide informed consent or if their clinical manifestations could be better explained by an etiology other than Zika virus infection (all infants were screened by a trained geneticist and serologic tests were performed for several other congenital infections). We also excluded mother-infant pairs when the mother had negative polymerase chain reaction assays for Zika in all specimens tested within 12 days from the beginning of her symptoms. Among the remaining patients, infants with CZS were selected as cases and infants without CZS, but with probable maternal Zika virus infection during pregnancy, were selected as controls.

### Data collection

Every recruited pregnant mother was evaluated by an obstetrician and an infectious disease physician. During this evaluation, we filled out a standardized questionnaire including the following demographic and pregnancy-related variables: maternal age (in years), income (in Brazilian minimum wages units), smoking status, alcohol and illicit drug consumption (if present or not), number of previous gestations, gestational week in which prenatal care had begun, and gestational age at symptom onset (based on an obstetric ultrasound at the first trimester). Pregnant women were serologically evaluated for HIV, syphilis, toxoplasmosis, rubella, and dengue. Serologic tests for Zika infection were not performed because they were either unavailable or unreliable at the time patients were recruited [12]. A real-time reverse transcription polymerase chain reaction (RT-PCR) for Zika was also performed for women referred to our clinic with recent symptoms (up to 12 days of symptoms).

Infants were evaluated by an infectious disease physician, a neurologist, and a geneticist. Every infant should have been assessed with:1) Zika virus RT-PCR on blood specimens, 2) laboratory evaluation for other congenital infectious etiologies including toxoplasmosis, syphilis, cytomegalovirus and rubella, and HIV, 3) a neuroimaging study, 4) an abdominal ultrasonography, 5) a brainstem evoked response audiometry (BERA), 6) an echocardiogram, and 7) a fundoscopy performed by an ophthalmologist. However, since some infants were referred at a later age, it was not always feasible to perform the complete evaluation.

In order to exclude other causes for the congenital abnormalities, all children were evaluated by an experienced geneticist to rule out genetic abnormalities. Other infections were ruled out as described; cytomegalovirus: a negative urine PCR in the first 21 days of life and/or the IgG clearance during the concept infancy period (without IgM reactive). Toxoplasmosis, rubella, and dengue infections: IgG clearance during the concept infancy period (without IgM reactive). Syphilis: non-treponemic test (VDRL) negative. HIV: serology test (ELISA) non-reactive.

### Statistical analysis

Data collected in the questionnaires were stored in a database using Access 2013® software. Subsequently, distributions of all variables were studied. The frequency of all of them have been described and comparisons between CZS cases and controls were performed using the Mann-Whitney test (continuous variables) or Fisher’s exact test (categorical variables). A power analysis was not performed, since the epidemic finished in Brazil, therefore we would not be able to enroll new patients.

STATA, v13.0 (Texas, USA) was used for all the statistical analysis.

### Ethical statement

This project and its informed consent were approved by the Ethics Committee of the IPPMG (approval number: 1.546.045). Written informed consent was obtained from all participants guardians. The guardians were approached during the paticipant’s first visit at the Infectious Diseases Clinic, if they accepted to participate, data were systematically collected during their clinical appointments.

## Results

### Sample characteristics

A total of 42 infants were included in our analysis, 18 controls and 24 cases. Infants were born between 12/11/2015 and 07/08/2017. Ten of them were born to mothers who had received antenatal care at our clinic, providing prospective information. The 32 remaining children were recruited either for being born with congenital abnormalities compatible with CZS or because their mothers had a history of symptoms compatible with Zika infection during pregnancy. The demographic characteristics of the sample are shown in Table 1.

**Table 1:** Maternal demographics between cases and controls

Maternal demographic and gestational variables	Total population (*n* = 42)	Controls (*n* = 18)	Cases (*n* = 24)	*P*-value
Week of gestation at time of symptoms, median (IQR^a^)	13 (8.25–24.25)	20	10	< 0,01
Maternal age at delivery in years, median (IQR)	21 (19.75–29)	26.5	23	0.95
Week of gestation at delivery, median (IQR) (missing data: 1)	38 (37–39)	39	38	< 0.02
Household income *per capita*^b^, median (IQR) (missing data: 15)	0.43 (0.25–0.71)	0.33	0.46	0.60
No. of previous gestations, median (IQR)	1 (0–3)	1	2	0.89
Week of gestation at first prenatal visit, median (IQR) (missing data: 14)	7 (6–15.25)	6.5	7.5	0.96
Substance use during pregnancy (%)				
Tobacco	6/40 (15.00%)	2/18 (11.11%)	4/22 (18.18%)	0.67
Alcohol	10/41 (24.39%)	4/18 (22.22%)	6/23 (26.09%)	1
Illicit drugs	2/40 (5.00%)	0/18 (0%)	2/22 (9.09%)	0.49
Maternal coinfections during pregnancy^c^ (%)				
Syphilis	3/34 (8.10%)	2/16 (12.50%)	1/21 (4.76%)	0.57
Human Immunodeficiency Virus	4/37 (10.81%)	3/16 (18.75%)	1/21 (4.76%)	0.30
Maternal dengue virus IgG reactivity at initial evaluation (%)	9/24 (37.50%)	5/12 (41.67%)	4/12 (33.33%)	1

^a^IQR, interquartile range

^b^Household income was presented in monthly Brazilian minimum wages, which is R$937 Brazilian reals (approximately $290 U.S. dollars)

^c^There was no evidence of toxoplasmosis coinfection among the 35 pregnant women tested

### Laboratory confirmation of maternal infection

PCR analyses for Zika in blood, urine or both were performed in 19 of the 36 mothers that reported a rash during pregnancy, and 11 (57.9%) tested positive in at least one specimen. Eight mothers with negative PCR results were not excluded either because they only had one specimen tested or because the PCR test was performed more than 12 days after symptoms onset. Among symptomatic women without a laboratory confirmation, the diagnosis of probable Zika infection was made based on clinical and epidemiologic information, as well as with the exclusion of other known causes (drug allergies or other infections).

### Infant assessment - evaluation of congenital anomalies

Twenty-one infants had microcephaly. One of them was born with a cephalic perimeter of 32 cm, but the head circumference stopped growing afterwards, and therefore was also considered as having microcephaly. Thirty-six children performed a neuroimaging exam. Among 6 children that were not evaluated with any neuroimaging exam, all of them were born with normal cephalic perimeter and were classified as controls since there were no other clinical or imaging abnormalities. Other components of the evaluation (fundoscopy, BERA, abdominal US and echocardiography) were performed in most infants. Among the children with CZS, fundoscopy was abnormal in 9 infants from a total of 21 who performed the exam; BERA was altered in 3 from a total of 17; 5 infants presented with patent foramen ovale, although at least 3 resolved in a subsequent echocardiography; 2 were born with clubfoot and 2 with cryptorchidism.

### Laboratory confirmation of CZS

PCR analyses for Zika in the blood, liquor or both were performed in only 11 (26%) of our infants. All of them yielded negative results. The median age at which the test was performed was 4 days.

### Association between occurrence of CZS and maternal variables

**Fig. 1:**
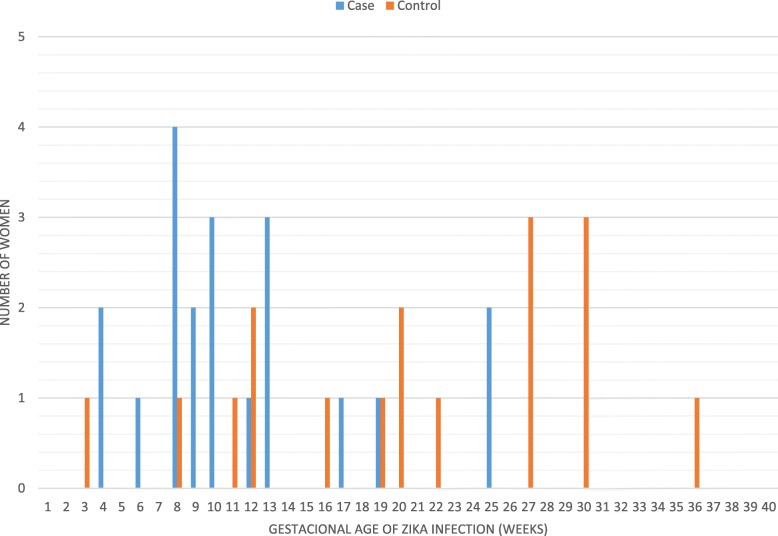
Maternal Gestational Age at Zika infection. Legend: Cases were defined as royal blue lines, and controls as orange lines

We tested the association between the presence of CZS and the following variables: maternal age, gestational age in which symptoms occurred, gestational age in which prenatal care had started, occurrence of maternal coinfections, history of previous dengue infection (IgG reactivity in the first maternal evaluation), and use of alcohol, tobacco, or illegal drugs during pregnancy (Table 1). Gestational age in which infection occurred was the only variable that showed a statistically significant association with the presence of the CZS. Earlier infection was positively associated with the development of CZS (odds ratio = 0.87, 95% CI: 0.78–0.97; *p* <  0.01). The Fig. 1 demonstrated the gestational age at the mother’s infection for cases and controls. Case’s mothers presented symptoms earlier than controls.

## Discussion

In our study, we were able to evaluate several maternal factors that would be associated with CZS among exposed infants. Lower gestational age at the maternal Zika infection was the main factor associated with CZS. Indeed, all mothers from infants classified as cases presented symptoms before the 25th week of gestational age, while controls presented them at any point during gestation. We also found a negative association between gestational age at delivery and the presence of CZS. This last finding is likely a result, rather than a cause, of the development of the CZS.

Evidence that earlier maternal infection during pregnancy is associated with a higher likelihood of development of congenital abnormalities in the infant has already been provided by case-control, cohort and modelling studies as well as by reports of national surveillance systems [6–8, 10, 11, 13–17]. The biological plausibility of this finding is supported by the knowledge that other congenital infections also show the same association between early gestational age during infection and incidence of congenital abnormalities.

The literature has a relative paucity of data on which other maternal factors may be associated with the development of CZS among exposed fetuses. Three studies have already tried to answer this question using a different design than ours. Ventura et al. conducted a case-control study to look for factors associated with the development of ophthalmologic abnormalities among infants deemed to have CZS with microcephaly. The only prenatal/maternal factor associated with ophthalmologic findings among children with microcephaly was the timing of gestational infection [11]. Halai et al. analyzed the outcomes of 123 gestations with laboratory evidence of Zika infection. They tested the association of congenital abnormalities with the maternal viral load, the severity of maternal symptoms at acute infection, as well as with the presence of maternal dengue virus IgG. No significant associations were found [13]. The third study, conducted by Santa Rita et al., compared mothers of neonates with and without microcephaly to assess which factors were associated with congenital anomalies [14]. The presence of a probable Zika infection during pregnancy was a study variable, not an inclusion criteria as it was in our study. The authors found a trend toward a higher prevalence of positive maternal dengue IgG and of maternal homemaker occupation among mothers of infants with CZS. The positive dengue IgG could represent a marker of increased exposition to *Aedes aegypti*. The association with the homemaker occupation may also be related to an increased exposition to mosquitoes, due to the known deposition of *Aedes* eggs in domestic water or poorer socioeconomical status, since the mother did not have an income [2, 3, 11].

Our study has two major limitations. First, it was an exploratory analysis of our clinical database. Since our sample size was not calculated to detect clinically significant differences in the variables evaluated, we could have missed small effect sizes if they existed. Secondly, due to logistical reasons, our study was limited by a significant amount of missing data, especially for the confirmation of infant and maternal Zika infection. The lack of laboratory confirmation of CZS in our cases does not seem so important given the epidemiological context and the exclusion of other known causes for the congenital anomalies. Likewise, the absence of a Zika virus RT-PCR test in 57.9% of the symptomatic mothers could lead to a misclassification bias. However, other causes of exanthematic disease were ruled out in our cohort. During our study period, Brasil et al. investigated 345 pregnant women with a cutaneous eruption in Rio de Janeiro, finding a positive Zika RT-PCR in 53% of tested women [10]. Thus, it is likely that most of the patients included in our study without laboratory confirmation were, indeed, infected by the Zika virus, considering the clinical context and the epidemiological situation in Rio de Janeiro at our study period.

Despite these limitations, our study brings two important contributions. First, it corroborates to the growing body of evidence indicating that the early moment of infection during pregnancy is a risk factor for the development of the CZS in the fetus. Secondly, we demonstrated that all cases of CZS occurred up to 25 weeks of gestational age, which should be confirmed or refuted in larger studies.

## Conclusion

Efforts and public health measures to decrease the consequences of Zika virus infection among pregnant women must be emphasized in the beginning of pregnancy, when those measures although more useful are more difficult to implement. Our study highlights the need for larger studies designed to identify risk factors for the occurrence of congenital abnormalities among infants born to infected pregnant women. The identification of reversible or avoidable risk factors could be of great public health importance.

## Abbreviations

BERA: Brainstem evoked response audiometry
CZS: Congenital Zika Syndrome
ELISA: Enzyme-linked immunosorbent assay
GA: Gestational age
HIV: Human immunodeficiency virus
IPPMG: Instituto de Puericultura e Pediatria Martagão Gesteira
PCR: Polymerase chain reaction
RT-PCR: Real-time reverse transcription polymerase chain reaction
SD: Standard deviation
US: Ultrasound
VDRL: Venereal disease research laboratory test
